# Periodontitis and Alzheimer's disease, common pathways in down syndrome

**DOI:** 10.3389/fdmed.2026.1930049

**Published:** 2026-09-07

**Authors:** Fabio Arriola-Pacheco, Alicia Leonor Pinzón-Té, Rodrigo Serrano-Piña, Bertha Arelly Carrillo-Ávila, Jaime Díaz-Zúñiga, Samanta Melgar-Rodríguez, Víctor Manuel Martínez-Aguilar

**Affiliations:** 1 Faculty of Dentistry, University of Toronto, Toronto, ON, Canada; 2 Faculty of Dentistry, Universidad Autónoma de Yucatán Mérida, Merida, Yucatán, México; 3 Department of Medicine, Faculty of Medicine, Universidad de Atacama, Copiapó, Chile; 4 Conservative Dentistry Department, Faculty of Dentistry, Universidad de Chile, Santiago, Chile

**Keywords:** Alzheimer's disease, cytokines, down syndrome, genes, inflammation, periodontitis

## Abstract

Down Syndrome (DS) involves widespread systemic issues, including a near-universal development of early-onset Alzheimer's Disease, driven by factors beyond Amyloid Precursor Protein (APP) over-expression, such as chronic neuroinflammation and endosomal dysfunction. High prevalence of periodontitis in this population acts as a significant source of peripheral inflammation that may accelerate this cognitive decline, necessitating further study into the bidirectional, pro-inflammatory links between systemic health and neurodegeneration in DS. This review analyzes the common pathophysiological pathways linking periodontitis and AD development within the DS population, evaluating how oral dysbiosis acts as a systemic driver of neurodegeneration. We synthesized current molecular, microbiological, and clinical evidence evaluating the bidirectional relationships between trisomy 21-induced immune dysfunction, severe periodontitis, and accelerated cognitive decline. Individuals with DS exhibit a heightened susceptibility to aggressive, early-onset periodontitis starting as early as age six. This chronic oral dysbiosis facilitates microbial translocation, allowing periodontal pathogens (e.g., *Porphyromonas gingivalis*) and their virulence factors to cross the blood-brain barrier via circulatory or trigeminal routes. In the central nervous system, these pathogens encounter microglial populations already genetically primed by trisomy 21. This induces an exacerbated M1 microglial phenotype response, triggering sustained neuroinflammation, defensive over-deposition of Amyloid-β (Aβ) plaques, and upregulation of GSK-3β, which accelerates Tau protein hyperphosphorylation. While bidirectional links are strongly indicated, current literature lacks robust longitudinal studies connecting periodontal, microbiological, and cognitive data to establish definitive causality. Early clinical intervention and management of gum disease present a critical therapeutic window to delay the onset and slow the progression of AD in this vulnerable population.

## Introduction

Down Syndrome (DS) represents the most prevalent neurological condition worldwide (1). Varying prevalences have been reported worldwide, with there being approximately 1 in 700 people born with DS; a figure that is on the rise, mainly in Western countries (2). Advances in medical symptom control, quality of life, and life expectancy have been beneficial to this population. Nevertheless, there are still many challenges to overcome in the likes of understanding differences among the genotypes, specific symptom control (e.g., cognitive decline), access to healthcare services, and social integration of these individuals in some parts of the world. DS is characterized by numerous phenotypical manifestations such as brachycephaly, muscle hypotonia, motor skill problems, reduced neuronal density, cerebellar hypoplasia, and congenital heart defects. The expressed phenotypes hold the possibility to trigger manifestations that affect different systems, such as the cardiovascular, musculoskeletal, immune, and neurological ones (3). Hypothyroidism, autoimmune diseases, obstructive sleep apnea, epilepsy, hearing and vision problems, haematological disorders (including leukaemia), recurrent infections, periodontitis and Alzheimer's Disease (AD) are all conditions that can potentially burden SD individuals. Current understanding of some of the enumerated conditions suggests there are common pathways amongst some of these (4–6).

DS individuals experience an innate gene imbalance due to their chromosomal alteration, which in turn can lead to the development of particular conditions. Genome imbalance is prominently related to Amyloid Precursor Protein (APP) overexpression, and it has been classically theorized that the presence of APP is responsible for the rapid onset of AD in DS, but contemporary insights suggest that ApoE-ε4, endosomal dysfunction and neuroinflammation may also play a role (7). On the point of inflammation, there is growing evidence that the presence of inflammatory molecules in peripheral circulation, which are derived from certain inflammatory conditions, such as chronic liver disease, inflammatory bowel disease, and periodontitis, can be a potential risk factor for neuroinflammation. This is relevant as periodontitis, a dysbiotic low-grade inflammatory disease, is prevalent in the DS population. The higher prevalence of PD in the DS population has been reported to be in part due to innate immunological dysfunction, but is also exacerbated by difficulties with adequate oral hygiene and access to oral care (8).

## Methods

### Justification

The coexistence of DS and the near-universal development of early-onset AD represents a critical challenge for public health and neurobiological research. Historically, this accelerated cognitive decline has been attributed primarily to the over-expression of the *APP* gene encoded on chromosome 21. However, emerging evidence suggests that amyloid overproduction alone may be insufficient to fully account for the phenotypic aggressiveness of neurodegeneration in this population (9). This highlights chronic neuroinflammation and endosomal dysfunction as important, secondary pathological pathways that could potentially participate in disease progression.

Despite the fact that individuals with DS exhibit a genetically determined, heightened susceptibility to aggressive and destructive periodontitis starting in early childhood, current literature has frequently treated oral dysbiosis and central neurodegeneration as isolated clinical entities (10). A critical knowledge gap persists regarding the systemic, potentially bidirectional molecular and microbiological pathways that interconnect oral health with central nervous system pathology. Specifically, current research lacks a comprehensive synthesis of how the hypothesized translocation of key periodontal pathogens —such as *Porphyromonas gingivalis* and their virulence factors— across the blood-brain barrier (BBB) might acts as a peripheral factor that interacts with M1 microglial populations, which are suggested to be genetically primed by HSA21 trisomy (11–13).

Therefore, analyzing the common pathophysiological pathways linking periodontitis and AD development within the DS population is both fundamental and relevant. This review aims to unify fragmented evidence across immunology, microbiology, and neurology, bwhile exploring its potential translational value. By elucidating how peripheral inflammation of oral origin may act as a systemic contributor to neurodegeneration, this article provides a biological foundation to evaluate a potential window of clinical opportunity: the early management of periodontal health as an accessible, non-invasive strategy to investigate its impacts on the secondary inflammatory factors linked to cognitive decline in this vulnerable population. Thus, the aim of this article is to analyze the proposed common pathways between AD and periodontitis development in individuals with DS.

### Formulation of question's review and specific objectives

Review question 1: What are the precise molecular and cellular mechanisms through which trisomy 21-induced immune dysfunction predisposes individuals with DS to aggressive, early-onset periodontitis?. To answer this question we determine the specific objective: to delineate the immunological links between trisomy 21-induced systemic immune dysregulation and the heightened susceptibility to aggressive, early-onset oral dysbiosis.

Review question 2: How do periodontal pathogens, specifically *P. gingivalis* and their associated virulence factors, translocate from the oral cavity and cross the blood-brain barrier to interact with genetically primed microglia?, and its specific objetive to map the microbiological and circulatory routes involved in the translocation of periodontal pathogens and their virulence factors across the BBB into the central nervous system.

Review question 3: Through which common pathophysiological pathways does chronic peripheral oral inflammation upregulate M1 microglial phenotypes, Aβ plaque deposition, and GSK-3β/Tau hyperphosphorylation in this population?. To answer this question we proposed the following specific objective: to evaluate the synergistic effect between peripheral oral inflammation and genetically primed M1 microglial responses in accelerating amyloid-beta (Aβ) deposition and Tau protein hyperphosphorylation.

Review question 4: What clinical evidence supports the management of periodontitis as a viable therapeutic window to delay the onset or slow the progression of AD in individuals with DS?. For this review question, we developed the following specific objective: to identify current gaps in longitudinal literature and propose clinical periodontal interventions as a strategic therapeutic window to mitigate accelerated cognitive decline in the DS population.

### Search strategy and selection criteria

This comprehensive narrative review is based on a literature search conducted across PubMed/MEDLINE, LILACS, and SciELO, selecting peer-reviewed studies published between 2010 and 2026 in English and Spanish. The search architecture utilized combinations of Medical Subject Headings (MeSH) terms and free-text keywords, adapted for each database. The core search string included: (“Down Syndrome” OR “Trisomy 21” OR “HSA21”) AND (“Periodontitis” OR “Periodontal Diseases” OR “Oral Microbiota” OR “Porphyromonas gingivalis” OR “Oral Dysbiosis”) AND (“Alzheimer Disease” OR “Cognitive Decline” OR “Neuroinflammation” OR “Amyloid-beta” OR “Tau Protein”.

To ensure methodological transparency and minimize selection bias, the study selection process was performed independently by two investigators (F. A-P, and J. D-Z) who screened titles, abstracts, and full-text articles based on predefined criteria. Discrepancies regarding study eligibility were resolved through consensus or via consultation with a third senior reviewer. Inclusion criteria comprised: (1) observational clinical cohorts, case-control studies, and cross-sectional investigations evaluating periodontal status or cognitive trajectories in individuals with DS; (2) mechanistic, *in vitro*, and animal models characterizing the pathophysiology of trisomy 21-induced immune dysregulation or oral-brain microbial translocation; and (3) systematic or state-of-the-art narrative reviews providing foundational frameworks. Exclusion criteria involved: (1) studies lacking a clear biological focus on the DS, AD, or periodontal triad; (2) conference abstracts, editorials, and non-peer-reviewed preprints; and (3) narrative reviews that did not employ the Scale for the Assessment of Narrative Review Articles (SANRA) methodology. Data extraction was systematically focused on variables including study design, population characteristics (DS vs. euploid controls), specific immunological markers, periodontal pathogen strains, and neurodegenerative pathways. The extracted data were qualitatively synthesized to address the specific objectives of this review.

## Results

### Genes over-expression associated with chromosome 21-trisomy

**Figure 1:**
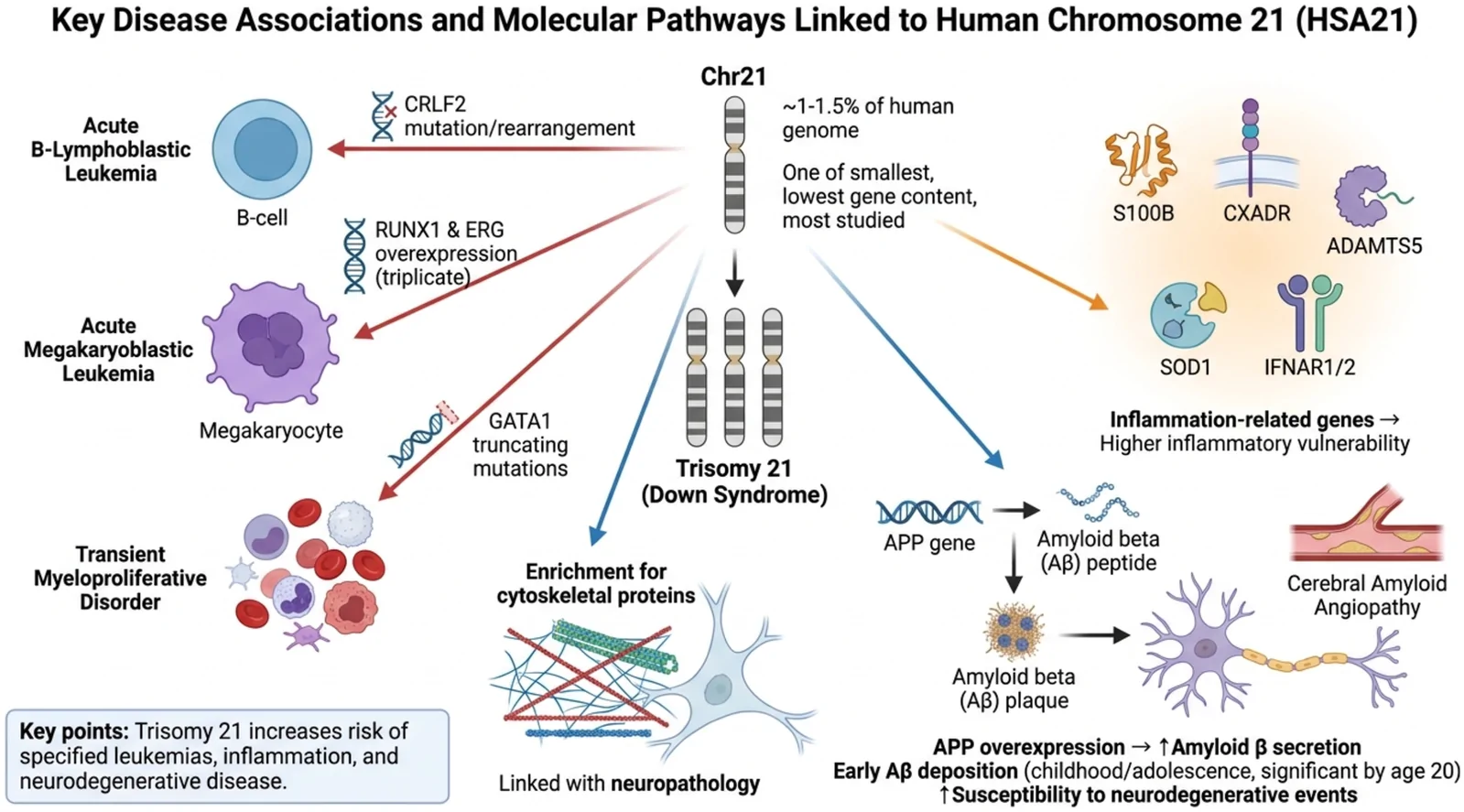
Genomic characteristics of human chromosome 21 (HSA21) and pathological implications in Down syndrome. Despite being one of the smallest chromosomes with the lowest gene content, HSA21 trisomy triggers a systemic molecular imbalance expressed through three main areas: **1) Hematological predisposition:** the over-expression of genes such as CRLF2, RUNX1, ERG, and mutations in GATA1 critically increase the risk of acute leukemias (B-lymphoblastic and megakaryoblastic) and transient myeloproliferative disorder. **2) Immune and structural regulation:** it hosts key inflammation-related genes (S100B, CXADR, ADAMTS5, SOD1, IFNAR1/2) and encodes cytoskeletal proteins linked to neurological disorders. **3) Early amyloid pathology:** APP gene over-expression drastically elevates beta-amyloid Aβ peptide secretion, driving the early deposition of extracellular plaques and cerebral amyloid angiopathy starting in childhood and adolescence.

The human chromosome 21 (HSA21) represents about 1%–1.5% of the human genome. While it is one of the smallest chromosomes, it remains one of the most extensively studied (Figure 1) (7, 14). Although considered to have a relatively low gene content, its imbalance is associated with multiple clinical phenotypes and pathologies, as observed in HSA21 trisomy (14). The trisomy is accompanied by an increased risk of developing Acute B-lymphoblastic leukaemia, which is often linked to mutations or rearrangements of Cytokine Receptor Like Factor 2 (*CRLF2)* or genes encoding other lymphocyte differentiation factors. Similarly, the increased risk of developing Acute megakaryoblastic leukaemia has been related to the over-expression of the *RUNX1* and ETS-related gene (*ERG)*, while transient myeloproliferative disorder is frequently associated with somatic truncating mutations in the GATA-binding factor 1 (GATA1) transcription factor gene (7, 15). Additionally, numerous inflammation-related genes are located on HSA21, including S100 calcium-binding protein B (*S100B)*, Ig-Like Cell Adhesion Molecule (*CXADR)*, a disintegrin and metalloproteinase with thrombospondin motifs 5 (*ADAMTS5)*, super oxide dismutase 1 (*SOD1)*, Interferon Alpha And Beta Receptor Subunit 1 and 2 (*IFNAR1*, *IFNAR2).* The over-expression of these genes represents a potential biological basis that may predispose individuals to inflammatory processes under physiological stress (16).

The HSA21 genotype provides a significant enrichment for encoded proteins located in cytoskeletal structures, which may be relevant given their reported involvement in neurological conditions and neuropathology (17). This genetic profile is closely linked to the over-expression of the *APP*, which is a key factor associated with the deposition of the cleavage peptide amyloid-beta (Aβ) in extracellular plaques and the potential development of cerebral amyloid angiopathy (7, 18). The over-expression of APP has been linked to increased Aβ peptide secretion, which may contribute to a higher susceptibility to neurodegenerative processes from early stages in life (19). Consistent with this biological rationale, individuals with DS can exhibit Aβ depositions during childhood and adolescence, with these deposits often becoming prominent by age 20 (20).

### Apoe expression and its potential role in down syndrome pathology

**Figure 2:**
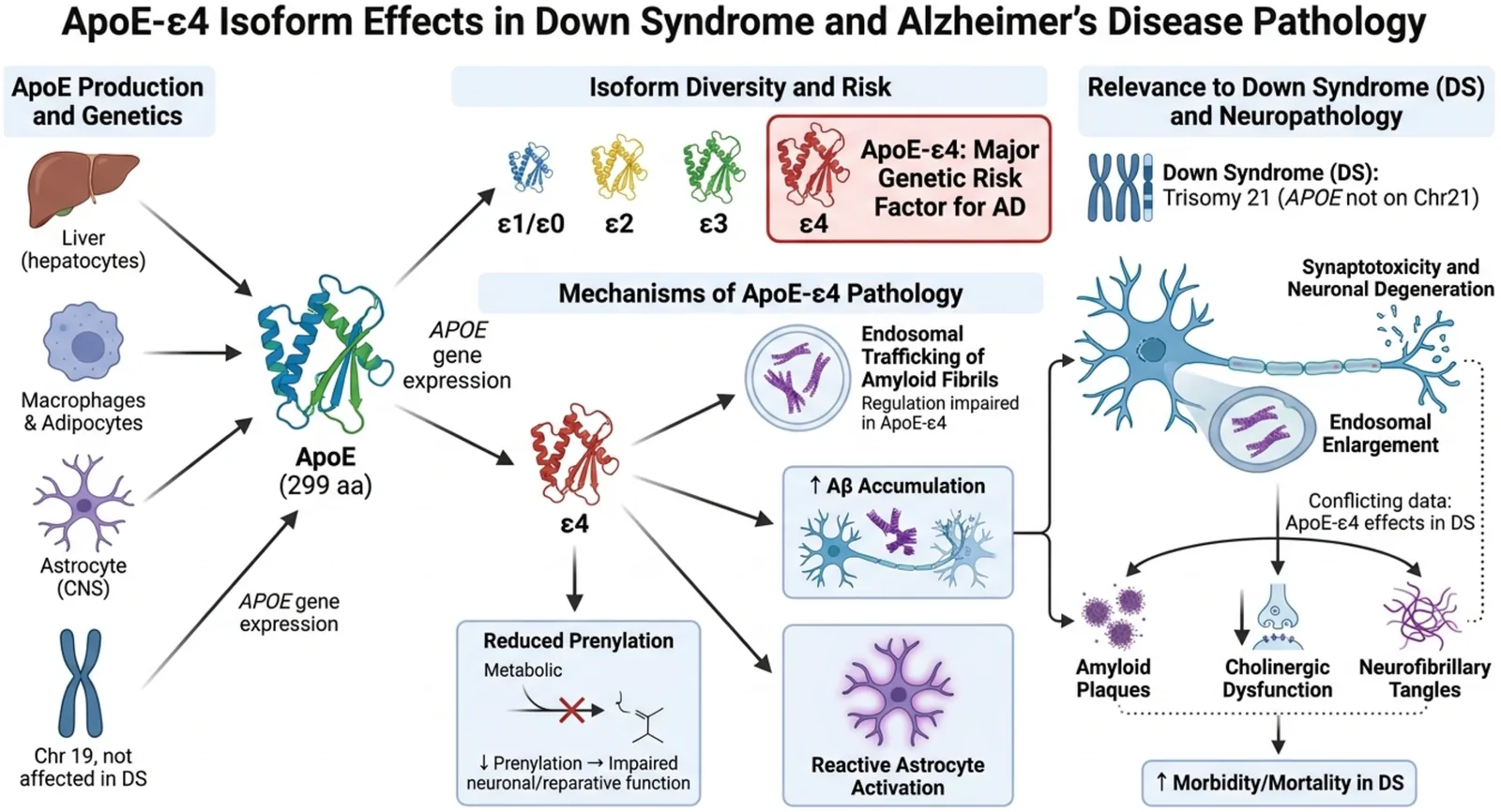
Role of the ApoE-ε4 isoform and endosomal dysfunction in Down syndrome neurodegeneration. Although the APOE gene (chromosome 19) is unaffected by trisomy 21, its ε4 isoform represents the highest genetic risk factor for Alzheimer's disease through three mechanisms: **1) Impaired repair and glial activation:** it possesses reduced prenylation capacity, which diminishes neuronal repair function and activates reactive astrocytes. **2) Amyloid and neurofibrillary burden:** it drives greater Aβ peptide accumulation, cholinergic dysfunction, and contributes to the development of Tau neurofibrillary tangles. **3) Endosomal synaptotoxicity:** it interacts with the intrinsic early endosomal enlargement of trisomic neurons by altering amyloid fibril trafficking, thereby exacerbating cellular degeneration.

ApoE is a 299-amino acid lipoprotein primarily produced by hepatocytes, macrophages and adipocytes in peripheral tissues, as well as by astrocytes in the central nervous system (CNS) (21). The *APOE* gene is located on chromosome 19; thus, its copy number is not directly affected by HSA21 trisomy. However, it remains highly relevant in these individuals, as the encoded protein is involved in synaptic function, maintenance, and tissue repair, typically showing increased activity under stressful or pathological conditions (Figure 2) (22). Among the four distinct ApoE isoforms, the *APOE* allele represents the strongest known genetic risk factor for AD (23). The ApoE-ε4 isoform exhibits a reduced prenylation capacity, which has been suggested to contribute to diminished neuronal or reparative function, enhanced induction of Aβ accumulation, and the activation of reactive astrocytes (22).

Data regarding the precise relationship between ApoE-ε4 expression and neuropathology in individuals with DS remain conflicting. Clinical and mechanistic studies suggest that the presence of the ApoE-ε4 is associated with amyloid burden and cholinergic dysfunction. This genotype has also been linked to increased morbidity and mortality in the DS population (24); as well as to elevated Aβ deposition (25); collectively, these factors are hypothesized to participate in the development of neurofibrillary tangles (26). Nevertheless, alternative or complementary mechanisms indicate that endosomal dysfunction —specifically early endosomal enlargement in trisomic neurons— may represent a contributing factor to synaptotoxicity and subsequent neuronal degeneration (27). This mechanism is highly relevant, as ApoE appears to regulate the endosomal trafficking of amyloid fibrils, and early endosomal enlargement has been documented in the brains of mice carring the ApoE-ε4 isoform (28).

### Immune dysregulation and inflammatory phenotype in down syndrome

**Figure 3:**
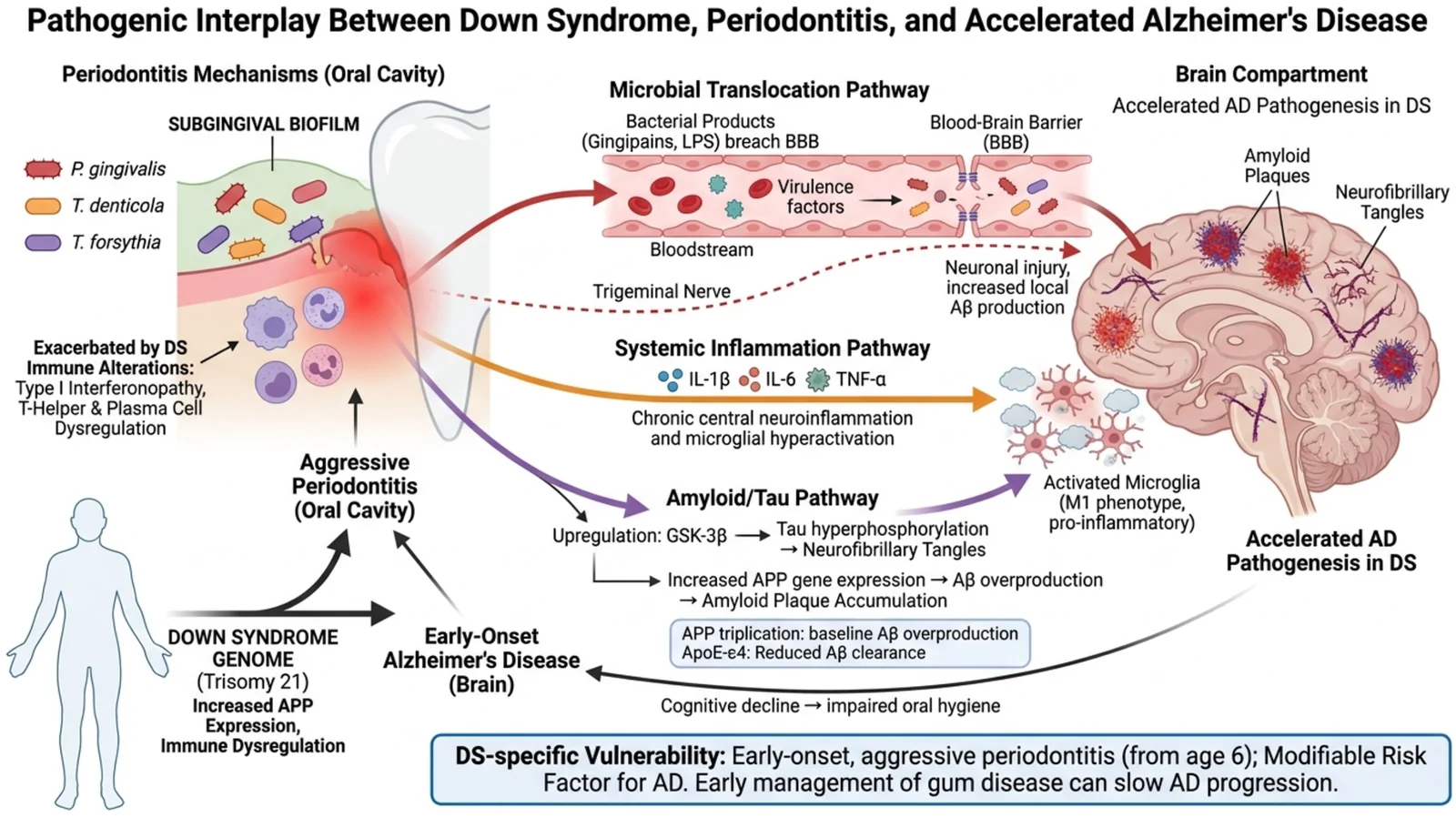
Immune deregulation and pro-inflammatory phenotype in Down syndrome (DS). Biological factors in DS drive a vulnerable systemic state through three core components: **1) Anatomical anomalies:** airway, ear, cardiac, and swallowing defects that facilitate early recurrent infections. **2) Cellular dysfunction:** impaired neutrophil/monocyte chemotaxis, abnormal NK cell function, and a decrease in naïve CD4 + T cells linked to thymic aberrations, alongside lower memory B cell response with altered immunoglobulin profiles (40% reduction in IgA). **3) Cytokine imbalance:** a selective increase in pro-inflammatory mediators (IL-1β, TNF-α, IFN-γ) that primes the system for systemic inflammatory response syndromes (SIRS) or sepsis.

The immune response in individuals with DS has been widely reported to be altered, with clinical manifestations often starting from a young age (29). Biological factors associated with the trisomy may predispose individuals with DS to a higher risk of developing chronic infectious diseases, potentially due to their characteristically pro-inflammatory genotype background (30). This genetic profile has been linked to anatomical variations, altered cytokine secretion patterns, and thymus abnormalities, which collectively may contribute to immune dysregulation (Figure 3). Anatomically, airway abnormalities, obstructive sleep apnea, congenital lower respiratory tract anomalies, congenital heart disease, congenital ear anomalies, and deglutition disorders represent potential factors that can contribute to a higher susceptibility to infections in this population (5, 31). Cellular function is also altered, with evidence indicating abnormalities in both primary and secondary immune responses. Amongst these, impaired neutrophil and monocytes chemotaxis has been suggested, which could potentially compromise tissue damage and infection control in these individuals (32). Furthermore, abnormal natural killer (NK) cell function has been reported, a phenomenon primarily linked to a higher count of these cells in individuals with DS compared to neurotypical controls (33, 34). Finally, a decrease in the number of naïve CD4 ^+^ T and CD8^ +^ T lymphocytes has been associated with anatomical aberrations of the thymus (35). Although a decrease in T lymphocyte counts has been detected in individual with DS as early as 5 years of age (36), B lymphocyte counts apparently do not exhibits significant age-dependent changes (37). This lower B-cell response, specifically involving impaired switched memory B cells, has been associated with inadequate antibody responses to vaccination (38). In this context, serum Immunoglobulin (Ig) A levels have been reported to be lower compared to controls (38), while IgG1 and IgG3 levels appear normal or higher, and IgG2 and IgG4 tend to present at lower concentrations (39).

In addition to cellular alterations, variations in cytokines secretion patterns have been described in the DS population, potentially affecting the activation and communication of key immunological elements. Compared to euploid individuals, individuals with DS have shown elevated levels of the pro-inflammatory mediators Interleukin (IL)-1β, tumor necrosis factor (TNF)-α, and Interferon (IFN)-γ, whereas IL-4, IL-6, IL8 and IL-10 have shown no significant differences between groups (40). These findings support the immunological phenotype hypothesis in DS, suggesting that this cytokine imbalance may participate in processes akin to systemic inflammatory response syndrome (SIRS) or compensatory anti-inflammatory response syndrome (CARS), potentially facilitating the development of sepsis in this population (29, 41). Characterizing these immunological parameters is highly relevant for contextualizing the shared pathways among different inflammation dependent pathologies.

### Microbiota interactions and dysbiosis in down syndrome

**Figure 4:**
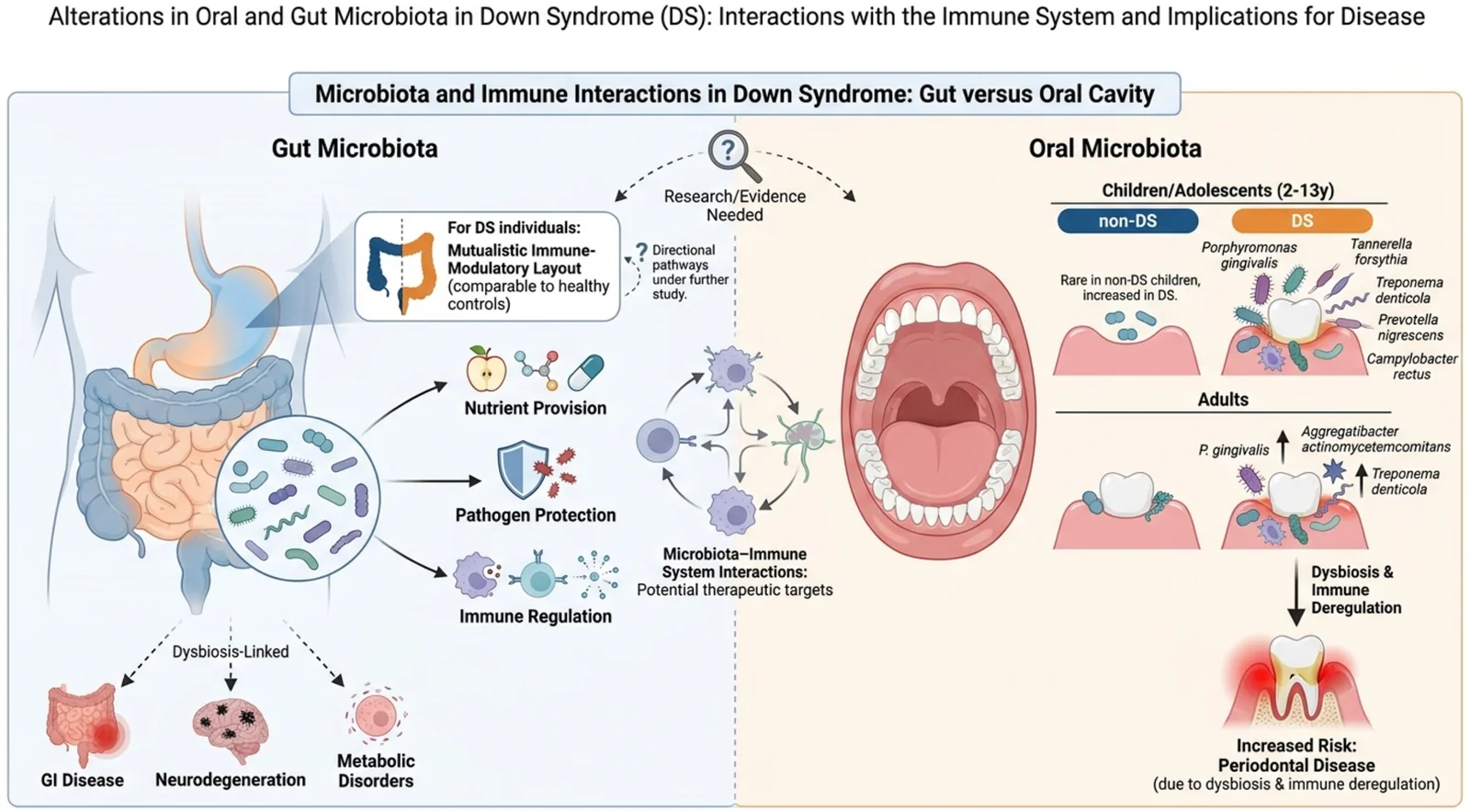
Microbiota composition and oral dysbiosis profiles in Down syndrome (DS). The interaction between a deregulated immune system and host traits disrupts microbial homeostasis, elevating periodontitis risk: **1) Gut microbiota:** maintains a mutualistic, immunomodulatory layout comparable to healthy controls. **2) Early oral dysbiosis:** children and adolescents (ages 2–13) exhibit atypical colonization of aggressive periodontal pathogens such as Porphyromonas gingivalis, Tannerella forsythia, and Treponema denticola. **3) Adult persistence:** high concentrations of P. gingivalis, T. denticola, and Aggregatibacter actinomycetemcomitans consolidate, driving accelerated and severe periodontitis onset.

Advances in the understanding of the human microbiome suggest that specific interactions between the microbiota and the immune system may represent potential targets for the management of certain pathological conditions (42). The oral and gut microbiomes host a vast number of microorganisms that are relevant to both homeodynamics and dysbiosis (Figure 4). The gut microbiota is hypothesized to play a role in nutrient provision, pathogen protection, and immune regulation; its dysbiosis has been associated with conditions such as chronic gastrointestinal diseases and neurodegenerative disorders (43). Existing studies on the gut microbiota of individuals with DS have shown an overall mutualistic, immune-modulatory profile that appears comparable to that of healthy controls (44), nevertheless further longitudinal studies are required to determine specific directional pathways and potential causal links.

The oral microbiota is likewise composed of a complex and dynamic network of microorganisms whose stability depends on both intrinsic microbial and host characteristics (45). *P. gingivalis* has been reported in higher concentrations in children and adolescents with DS compared to those without the condition (46). Furthermore, the presence of *Tannerella forsythia, Treponema denticola, Prevotella nigrescens*, and *Campylobacter rectus* has been documented in individuals with aged 2–13 years, an occurrence that is typically infrequent within this age group in non-trisomy cohorts (47). In adult populations, higher levels of *P. gingivalis*, *Aggregatibacter actinomycetemcomitans* and *T. denticola* have been reported in subjects with DS compared to non-DS controls (48, 49). These variations in the detection of different periodontal pathogens, when considered alongside the characteristic immune dysregulation of DS, suggest that this population may exhibit a heightened susceptibility to developing oral dysbiotic condition, such as periodontitis.

Mechanistic models, primarily derived from animal and *in vitro* studies, suggest that the chronic presence of these periodontal pathogens and their virulence factors, such as lipopolysaccharides and gingipains, may induce a state of chronic low grade inflammatory phenotype (CLIP) (50–52). In the context of DS, it is hypothesized that this peripheral inflammatory challenge could potentially interact with an already primed immune system, thereby representing a plausible pathway that might influence remote organs, including the central nervous system. However, while this oral-brain axis provides a compelling biological rationale, robust longitudinal data directly demonstrating these directional kinetics and their clinical consequences in the DS population remain limited.

### Down syndrome and Alzheimer's disease

**Figure 5:**
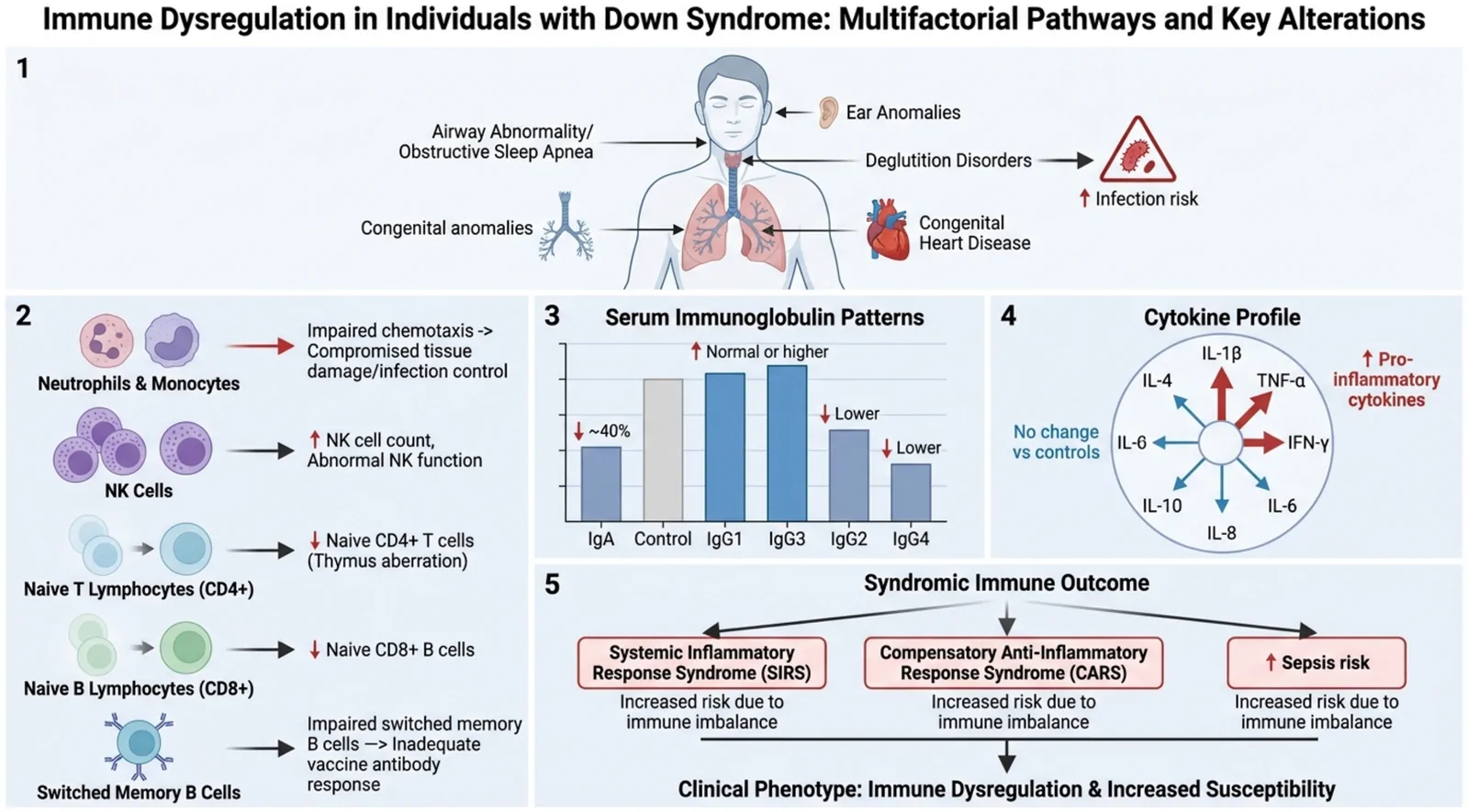
Neurogenetic and immune cascades in the development of Alzheimer's disease (AD) in Down syndrome (DS). AD pathogenesis in DS is a lifelong, progressive process beginning in prenatal stages through three main axes: **1) Genetic and endosomal drivers:** APP gene triplication and DYRK1A over-expression accelerate amyloidogenic processing, endosomal dysfunction, and early \(A\beta\) accumulation. **2) Interferonopathy and chronic inflammation:** the triplication of interferon receptor genes on chromosome 21 establishes a persistent systemic pro-inflammatory phenotype that hyper-activates microglia into a neurotoxic M1 state, raising cytokines (IL-1β, TNF-α) that drive Tau phosphorylation. **3) Biphasic trajectory and ferroptosis:** microglia exhaust their initial neuroprotective capacity under chronic oxidative stress, shifting into a locked, dystrophic phenotype that drives synaptic pruning, neurovascular damage, and iron-dependent cell death via ferroptosis.

A robust genetic foundation links DS to AD; nevertheless, this relationship warrants exploration beyond primary genetic drivers, as multiple secondary factors, such as systemic inflammation and microbiota dysbiosis, may potentially interact with and exacerbate the already vulnerable systemic phenotype (Figure 5). The AD-related neuropathology observed in the DS population has been associated with neurodevelopmental variations and altered cognitive trajectories (53). Extensive evidence suggest that a significant proportion of adults with DS over the age of 40 exhibit neuropathological changes consistent with AD, including the deposition of Aβ peptide in diffuse and neuritic plaques (16). Furthermore, a high prevalence of clinical dementia has been documented in individuals with DS by their late 60s, a phenomenon widely hypothesized to be linked to the characteristic *APP* over-expression accompanying the trisomy (19). Additionally, preliminary studies suggest that early neuropathological features may be observable from gestational stages, including potential variations in neurogenesis, synaptogenesis, and myelination in DS fetuses (54).

The characteristic pro-inflammatory genetic background in individuals with DS may also represent a contributing factor to AD neuropathology, potentially by promoting neuroinflammatory states that could stimulate pro-inflammatory microglial activation and subsequent cytokine secretion (55). This mechanism has been explored through observed associations between plasma IL-1β levels and phosphorylated Tau protein in individuals with DS (56). This link is relevant because IL-1β has been suggested to participate in aberrant glutamate release —a condition that, within the context of neurodegenerative processes, may contribute to cellular damage and foster molecular environments associated with AD onset (57). Within this inflammatory network, microglia-derived IFN-γ has been reported to stimulate an increase in M1 subset cytokines, such as IL-1β, IL-6, IL-12, IL-23 y TNF-α, alongside the inhibition of IL-10 and the over-expression of APP, thereby potentially sustaining a chronic pro-inflammatory state in individuals with DS (58–60). The neuropathological effects of dual-specificity tyrosine-regulated kinase 1A (*DYRK1A)* and endosomal dysfunction represent additional mechanisms proposed to clarify the AD-DS relationship (3). In animal models, *DYRK1A* over-expression has been observed to phosphorylate APP by Thr 668 (61). This phosphorylation is hypothesized to facilitate the amyloidogenic processing of APP by β-secretase and γ-secretase, potentially leading to increased Aβ production (62). Collectively, these proposed mechanisms complement currently known pathways and highlight the relevance of exploring these specific pathophysiological networks.

In general terms, the neuroinflammatory cascade associated with AD in individuals with DS is conceptualized as a lifelong, progressive process. This cascade is hypothesized to initiate with early immune dysregulation, is suggested to be modulated by genetic interferon over-expression, and is characterized by a proposed transition phase involving microglial exhaustion and iron-dependent cellular death (63–66). The shift toward “Inflammation-first” pathological frameworks has significantly modified the proposed temporal timeline of DS-AD. Historically viewed as a secondary response to Aβ deposition, chronic neuroinflammation is increasingly recognized as a potential upstream contributor that may precede significant plaque accumulation (64, 67). In individuals with DS, microglial activation and astrogliosis have been documented in the frontal and temporal cortices as early as the late twenties and early thirties, representing a potential priming mechanism for subsequent tau phosphorylation and synaptic loss (55, 68). Furthermore, lifelong and prenatal immune dysregulation appear highly relevant; emerging multi-omics research and brain atlas studies have detected molecular signatures associated with neurodegeneration and systemic immune dysregulation shortly after birth (ages 0–3) (65, 66). This indicates that trisomy 21 imprints a lifelong, altered immune phenotype. Rather than acting as a late-onset geriatric complication, the neuroinflammatory landscape in DS appears to develop over a lifespan, potentially creating an vulnerable neural environment decades before clinical dementia manifests (63, 69).

The proposed biphasic microglial phenotype represents another key factor to consider (70). Recent preclinical models suggest that microglial behavior in DS follows a distinct biphasic trajectory. During the early phase, microglia are hypothesized to initially adopt a highly active, neuroprotective state in attempt to clear early metabolic stress and maintain redox homeostasis. Conversely, during the late phase, chronic exposure to trisomy 21-induced oxidative stress is hypothesized to contribute to the exhaustion of their protective capacity (71, 72). Consequently, the cells may shift into a restricted, hyper-inflammatory, and dystrophic phenotype that could participate in synaptic pruning and contribute to neurodegenerative pathways. Furthermore, the genetic interferonopathy and peripheral drivers factors appear to act as relevant drivers. The triplication of chromosome 21 is linked to a permanent over-expression of four critical interferon receptor genes (*IFNAR1, IFNAR2, IFNGR2,* and *IL10RB*), which has been associated with a chronic state of Type I interferonopathy (73–75). This peripheral immune system hyper-reactivity may continually signal to the central nervous system. Coupled with blood-brain barrier (BBB) leakage, this peripheral-central crosstalk is hypothesized to amplify the brain's internal inflammatory response and potentially contribute to progressive white matter variations (76). Finally, ferroptosis and vascular reversibility may also play a vital role in this network. Emerging evidence points to ferroptosis —an iron-dependent, non-apoptotic form of cell death— as a potential source of early oxidative stress in the DS brain (77). Concurrently, novel structural imaging has shown that early neurovascular lesions and white matter hyperintensities in young adults with DS exhibit unexpected fluctuations (77, 78). This suggests a potential window of therapeutic oportunity, where addressing vascular inflammation at an early stage might help to modulate or delay permanent structural variations.

### Periodontitis and Alzheimer's disease, pathways in down syndrome

As previously outlined, the intrinsic characteristics of DS exhibit a close association with the development of inflammatory and dysbiotic conditions, such as AD and periodontitis. The relationship between AD and periodontitis has been extensively explored in the literature (79), with evidence suggesting that oral dysbiosis may participate in the translocation of oral pathogens, such as *P. gingivalis and T. denticola,* to extraoral sites including the brain. Furthermore, the local inflammation associated with periodontitis is hypothesized to contribute to systemic inflammatory states, thereby potentially playing a role in neuroinflammatory AD pathways. Within this framework, the molecular and genetic characteristics of DS that alter normal physiological homeodynamics are hypothesized to be vulnerable to exacerbation by comorbid conditions, such as periodontitis. This baseline inflammatory state may also influence susceptibility to more severe forms of periodontitis, which in turn could represent a contributing factor to neuroinflammation, neurodegeneration, and altered cognitive trajectories (80). The genotypic variations, alongside potential defects in T-helper lymphocyte subset activation and plasmacytic function, are hypothesized to favor a chronic pro-inflammatory background. This environment may increase the susceptibility to subgingival microbiota dysbiosis, potentially modulating both local and systemic inflammatory responses. Once systemic inflammation pathways are active, periodontal bacteria, their virulence products, and locally and systemically induced cytokines may potentially reach the central nervous system and amplify internal pro-inflammatory responses (81). The M1 microglial phenotype might exhibit an enhanced response due to already primed genetic factors in DS, representing a plausible pathway that could participate in neuroinflammatory responses, Tau protein phosphorylation, and the progression of AD-related pathology (60, 80). The presence of inflammatory mediators associated with M1 activation has been linked to increased *APP* expression, potentially contributing to elevated Aβ secretion. Concurrently, diminished clearance mechanisms associated with the ApoE-ε4 isoforms could contribute to greater Aβ accumulation (82). These interrelated mechanisms should be considered alongside the baseline genetic predisposition of individuals with DS to develop AD neuropathology due to *APP* over-expression and potential endosomal dysfunction. Broadly considered, bidirectional hypotheses suggest that periodontitis might represent a contributing factor to AD progression from earlier stages of life, or conversely, that individuals with DS experiencing AD-related changes may exhibit a higher susceptibility to early-onset periodontitis. Consequently, in the DS population, both periodontitis and AD-related features may present at earlier ages and potentially display greater progression and severity compared to neurotypical controls.

**Figure 6:**
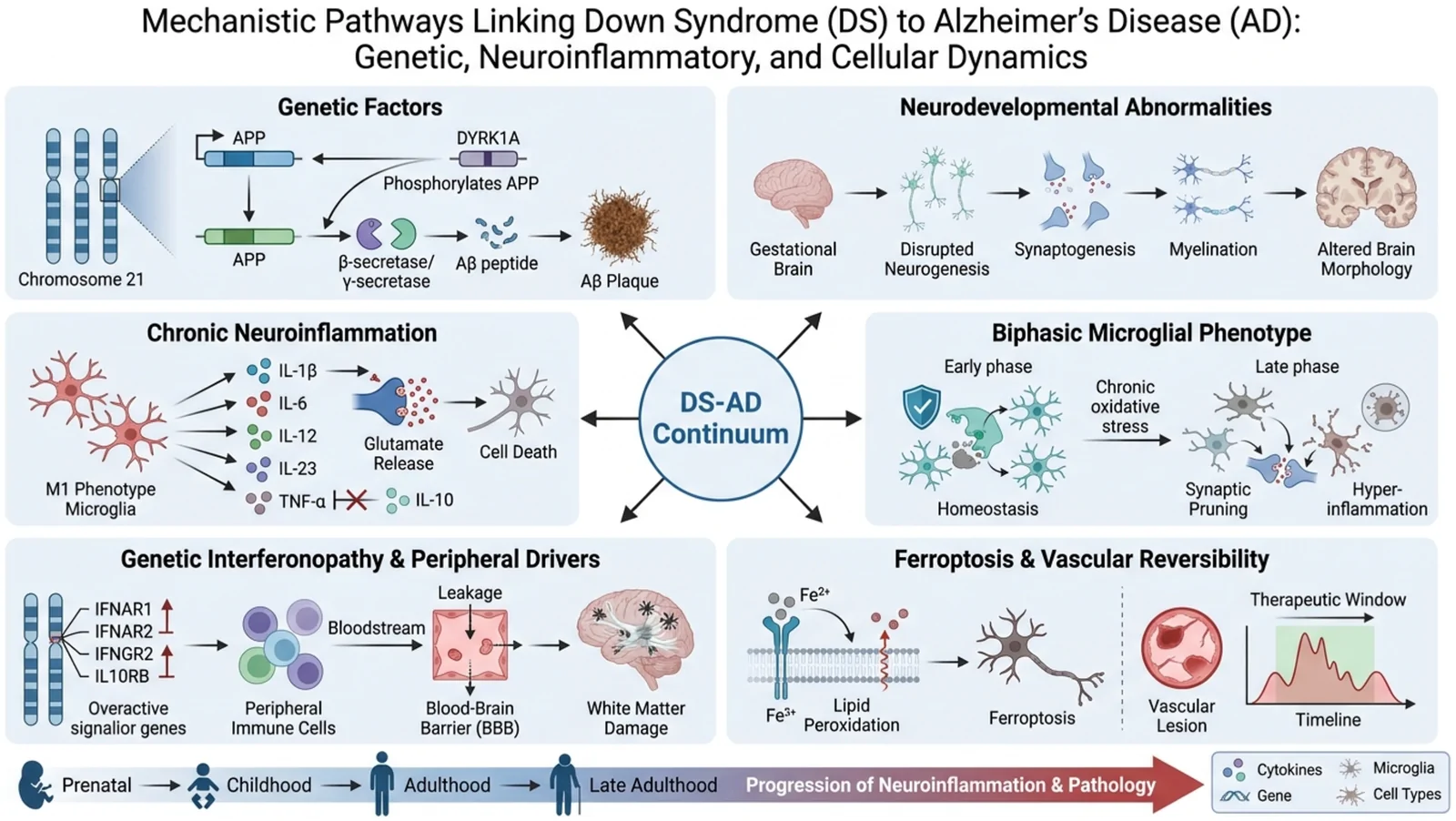
Pathogenic mechanisms linking periodontitis and Alzheimer's disease (AD) in Down syndrome (DS). Periodontitis acts as a critical environmental accelerator of neurodegeneration in DS through three interconnected pathways: **1) Microbial translocation:** oral pathogens and their toxins (such as P. gingivalis) migrate to the brain via blood or neural routes, damaging the blood-brain barrier. **2) Amplified neuroinflammation:** systemic inflammation hyper-activates microglia (M1 phenotype), which are already genetically primed by trisomy 21. **3) AD exacerbation:** this pro-inflammatory environment accelerates Aβ plaque deposition and Tau protein hyperphosphorylation, speeding up cognitive decline. Early management of periodontitis represents a key clinical window to slow down this progression.

Indeed, periodontitis is hypothesized to represent a relevant environmental factor that may interact with the progression of AD-related pathology in individuals with DS (80, 83). While the genetic over-expression of *APP* on chromosome 21 establishes a primary biological driver for early AD neuropathology, the highly aggressive, early-onset periodontitis frequently observed in DS is suggested to create a state of continuous peripheral inflammation that could potentially participate in the acceleration of neurodegenerative processes (80, 83). The proposed biological pathways connecting periodontitis, DS, and AD encompass several interconnected mechanisms (Figure 6). Microbial translocation represents a primary potential pathway of connection. Proposed mechanisms indicate that the trigeminal nerve and hematogenous routes may allow key periodontal pathogens —primarily *P. gingivalis*, *T. forsythia*, and *T. denticola—* to translocate from the oral cavity into the central nervous system (84). Preclinical models suggest they may achieve this by invading the bloodstream or migrating along the trigeminal nerve architecture (85). Additionally, virulence factors such as gingipains and lipopolysaccharides secreted by *P. gingivalis* cross into the brain (51, 86–88). In experimental models, these bacterial products have been associated with structural neuronal alterations, the degradation of tight junction proteins within the BBB, and induction of local Aβ accumulation (51, 85, 89, 90).

Sustained systemic and central neuroinflammation is hypothesized to contribute as a shared pathological pathway. Within this framework, a proposed “vicious cycle” of microglial activation may initiate when oral pathogens or their systemic inflammatory mediators (such as IL-1β, IL-6, and TNF-α) reach the central nervous system, encountering a microglial population that is suggested to be genetically primed by HSA21 trisomy (91). Preclinical evidence suggests that this interaction may stimulate an aggressive, chronic innate immune response. Consequently, the continuous inflammatory challenge associated with severe periodontitis is hypothesized to facilitate the transition of microglia from a neuroprotective phenotype to a restricted, hyper-inflammatory, and dystrophic state, potentially participating in aberrant synaptic pruning and contributing to cognitive decline (60, 92–95).

Additionally, emerging hypotheses suggest that the brain may produce Aβ partly as an antimicrobial peptides in response to microbial challenges, including oral spirochetes. Because individuals with DS exhibit a baseline overproduction of Aβ due to *APP* gene triplication, periodontitis-associated microbial challenges could promote a defensive, elevated deposition of amyloid plaques (96–99). Furthermore, intracellular signaling cascades triggered *in vitro* by gingipains and microglial activation are suggested to upregulate glycogen synthase kinase-3 beta (GSK-3β)—a key enzyme associated with the hyperphosphorylation of Tau proteins—thereby potentially contributing to the formation of neurofibrillary tangles (96, 98, 99).

Finally, a DS-specific vulnerability window has been proposed in the literature, reflecting the frequently aggressive and early onset of periodontitis in this population. Unlike the typical clinical course in the general population, where periodontal disease prevalence often correlates with advanced age, individuals with DS can manifest aggressive forms of periodontitis starting as early as 6 years of age, a phenomenon linked to intrinsic immune variations (80). In this context, the chronic, peripheral Type I interferonopathy characteristic of DS is hypothesized to lower the threshold for systemic immune disruption, potentially causing minor oral dysbiosis to generate a elevated systemic inflammatory response (80). Collectively, these findings suggest that periodontitis represent an important, modifiable risk factor. Investigating the early management of periodontal disease offers a potential window of clinical opportunity to evaluate whether modulating oral dysbiosis can help manage the secondary inflammatory factors associated with AD progression in the DS population (80).

### Critical evaluation of the Oral-Brain axis in Down syndrome: contradictions and alternative pathophysiological explanations

A rigorous and balanced evaluation of the oral-brain axis in DS requires a comprehensive analysis of the profound methodological discrepancies, replication failures, and alternative genetic mechanisms that challenge the direct causal link between periodontitis and AD. Although the biological plausibility interconnecting oral dysbiosis with central neurodegeneration is compelling, the field remains highly polarized due to conflicting empirical evidence (Table 1).

**Table 1:** Categorization of pathophysiological evidence and levels of validation.

Mechanism/pathway	Specific evidence in DS populations	Extrapolated from general/sporadic AD	Inferred from experimental/animal models
Genetic Driver & Overexpression	High. Documented *APP* and *DYRK1A*triplication, early Aβ deposition by age 20.	*Not applicable (specific to DS genetic architecture).*	Validated in trisomic mouse models (e.g., Ts65Dn).
Oral Microbiota Dysbiosis	Moderate. Higher prevalence of *P. gingivalis* and spirochetes in children/adults with DS.	Well-characterized link between severe periodontitis and sporadic AD risk.	Mechanistic tracking of oral-hematogenous translocation pathways.
Systemic & Neuroinflammation	High. Chronic pro-inflammatory cytokine profile (IL-1β, TNF-α) and Type I interferonopathy.	Chronic peripheral inflammation correlates with accelerated cognitive decline.	Microglial priming and accelerated synaptic pruning dynamics.
Biphasic Microglial Exhaustion	None/Theoretical. Not yet validated in living human DS tissue.	Observed morphological changes in late-stage post-mortem AD brains.	High. Documented in recent preclinical models of chronic oxidative stress.
Ferroptosis & Vascular Reversibility	Low. Limited to initial structural neuroimaging showing WMH fluctuations).	Iron dysregulation and microvascular lesions documented in advanced AD.	High. Characterized as a major source of early oxidative destruction *in vitro*.

First, several independent studies have failed to detect periodontal pathogens in AD brains, directly contesting the universality of the infectious hypothesis. While pioneering cohorts historically identified *P. gingivalis* DNA and its toxic proteases (gingipains, specifically RgpB and Kgp) in a high percentage of post-*mortem* human cerebral tissues (51, 86), subsequent rigorous replication attempts by distinct international research groups have yielded negative, ambiguous, or negligible results. These localized or undetectable microbial signatures raise a critical epistemological question: is the cerebral infiltration of oral pathogens a mandatory upstream driver of AD pathogenesis, or is it an occasional, localized phenomenon restricted to specific sub-cohorts with extreme barrier permeability? The lack of standardized protocols for PCR amplification and immuno-histochemistry in post-*mortem* brain tissue further confounds these findings, as issues regarding sample contamination, post-*mortem* degradation, and tissue fixation techniques frequently obscure whether these microbes truly resided in the neural parenchyma during life.

Consequently, intense controversies regarding the role of *P. gingivalis* in AD remain at the forefront of neurobiological debates. Skeptics of the pathogen-induced model strongly argue that the presence of *P. gingivalis* or other periopathogens (such as *T. denticola* or *T. forsythia*) in the central nervous system represents a late-stage, opportunistic epiphenomenon rather than an etiological trigger. According to this “compromised barrier” narrative, advanced neurodegeneration inherently induces systemic immunosenescence, severe autonomic dysfunction, and a profound disruption of both the intestinal and blood-brain barriers (100). As the structural integrity of the BBB decomposes due to localized astrocytic and pericytic degradation, the weakened neural parenchyma becomes highly vulnerable to the passive infiltration of any circulating microorganisms or their circulating virulence factors (such as lipopolysaccharides and peptidoglycans) (101). Under this premise, oral pathogens do not initiate the cascade; they merely colonize an already decaying microenvironment, flourishing opportunistically due to the rich availability of metabolic substrates provided by damaged neural tissue.

Furthermore, there are robust and entirely alternative explanations for neuroinflammation in DS independent of periodontitis, driven by the intrinsic genetic architecture of trisomy 21 (16, 102). The triplication of chromosome 21 establishes a lifelong, sterile pro-inflammatory baseline that primally reshapes the central immune landscape long before the clinical onset of periodontal disease (102, 103). Key genes over-expressed in DS, such as *DYRK1A* and regulator of Calcineurin 1 (*RCAN1*), directly impair endosomal trafficking and accelerate tau phosphorylation independently of external stimuli (104). Crucially, chromosome 21 encodes the gene cluster for *IFNAR1, IFNAR2, IL10RB*, and *IFNGR2*. This genetic triplication leads to a chronic state of interferon hypersensitivity, which continuously “primes” microglia toward a highly reactive, hypersensitive M1-like phenotype (105). These genetically primed microglia exhibit an exaggerated, autonomous neuroinflammatory response, chronically secreting TNF-α, IL-1β, and IL-6 (106). This state of permanent neuroinflammation and subsequent accelerated deposition of Aβ occurs seamlessly in the absence of any peripheral microbial translocation, suggesting that oral dysbiosis may, at most, act as an aggravating peripheral amplifier rather than an indispensable primary cause.

These conceptual debates are further complicated by the severe limitations of animal models and post-*mortem* studies that dominate current literature. Translational research heavily relies on murine models to demonstrate that oral infection leads to cerebral amyloidosis; however, these models possess inherent flaws. Rodent studies typically employ acute, massive, and highly unphysiological oral inoculations of *P. gingivalis* over a span of weeks, forcing an artificial state of systemic bacteremia. This methodology completely fails to replicate the human clinical reality of DS, which involves a slow, low-grade, chronic exposure to subgingival biofilms spanning three to four decades. Conversely, human *post-mortem* tissue studies offer only a static, terminal snapshot of the disease at its end-stage. They are fundamentally incapable of establishing temporal sequences or directional causality. From a static post-*mortem* sample, it is scientifically impossible to determine whether the periodontal pathogens accelerated the cognitive decline, or if the cognitive decline led to severe self-care deficits, poor oral hygiene, and a subsequent exacerbation of aggressive periodontal disease.

Finally, there are profound, near-insurmountable challenges in proving oral-brain microbial translocation in humans during life. Demonstrating the active migration of *P. gingivalis* or its outer membrane vesicles from the subgingival pocket into the cerebral cortex via hematogenous dissemination or anatomical routes (such as retrograde transport along the trigeminal or olfactory nerves) cannot be ethically or technically performed in living patients. Current neuroimaging modalities (such as PET or functional MRI) can track active microglial neuroinflammation but lack the resolution to identify specific bacterial etiologies. Similarly, liquid biopsies of cerebrospinal fluid offer highly diluted biomarkers that rarely provide a definitive real-time tracking of microbial trafficking across the BBB. Without robust, multi-decade longitudinal human cohorts that pair continuous, quantitative microbiological mapping of the oral cavity with serial cerebrospinal fluid biomarkers and advanced high-resolution neuroimaging from early childhood through to the adult onset of dementia, the definitive causal validation of the oral-brain axis in the DS population remains suggestive and associative, rather than conclusively established.

### Clinical implications, healthcare barriers, and translational relevance

From a translational perspective, evaluating whether periodontal therapy can effectively modulate or delay the progression of AD-related cognitive decline in the DS population represents a crucial clinical frontier. However, a critical caveat must be acknowledged within the current scientific literature: to date, no interventional clinical trials exist assessing the longitudinal impact of periodontal treatment on cognitive outcomes or later dementia risk specifically within DS cohorts. While small-scale interventional studies in the general sporadic AD population have shown that periodontal scaling and root planing can reduce systemic inflammatory biomarkers (such as serum IL-1β and TNF-α), data definitively demonstrating a subsequent stabilization of cognitive decline remain limited and entirely absent for the trisomic population (107). Consequently, clinicians must frame early periodontal therapy as a validated means to preserve systemic health and masticatory function, rather than an explicit prophylactic guarantee against future cognitive decline.

Despite the lack of direct interventional efficacy data, the high prevalence and early onset of aggressive periodontitis in DS justify the immediate implementation of proactive, lifelong oral screening and preventive strategies. Given that aggressive, early-onset periodontitis can manifest in this population as early as age six, childhood prevention represents the foundational pillar of intervention (108). At this early stage, therapeutic success relies heavily on caregiver-assisted oral hygiene. Due to the variable degrees of cognitive and motor limitations inherent to trisomy 21, independent brushing is frequently insufficient to disrupt the aggressive subgingival biofilms characteristic of oral dysbiosis. Therefore, establishing structured training programs for parents and caregivers is essential to ensure meticulous, daily plaque control and to mitigate the early systemic inflammatory burden.

Concurrently, a rigorous protocol of periodontal monitoring must be established from pediatric dentistry through adulthood. Clinicians should transition from a reactive treatment model to a rigorous preventive protocol, establishing bi-annual or tri-annual professional periodontal evaluations that incorporate the longitudinal assessment of inflammatory biomarkers and microbiological shifts where feasible. Practical recommendations for multidisciplinary teams include utilizing localized antimicrobial adjuncts alongside meticulous chemical and mechanical biofilm control tailored to the patient's manual dexterity (109).

Finally, ensuring continuity of care requires a seamless transition to adult dental care. As patients with DS age, they often face a clinical vacuum when moving from pediatric clinics to general adult dentistry, a transition frequently complicated by escalating behavioral or medical complexities. Transition protocols must be structured and multidisciplinary, involving pediatricians, periodontists, and neurologists. Maintaining tight periodontal control into adulthood is vital, as this is the period when both severe tissue destruction and accelerated Aβ deposition intersect. However, translating these recommendations into clinical reality faces profound systemic barriers to oral healthcare inherent to the DS population. Individuals with DS frequently encounter severe cognitive, communication, and behavioral challenges that hinder cooperation during conventional dental procedures, often necessitating advanced behavioral management techniques or intravenous sedation, which severely limits care access (110). Furthermore, there is a critical shortage of specialized dental professionals trained to manage adult patients with complex neurodevelopmental conditions (111). This professional scarcity is compounded by socioeconomic barriers, including inadequate public healthcare coverage for comprehensive adult periodontal care, physical accessibility limitations in standard clinics, and a historical lack of integrated medical-dental guidelines (111). Addressing these structural barriers is a mandatory prerequisite to effectively evaluating the translational validity of the oral-brain axis and improving the quality of life and neurological health trajectories of individuals with DS.

## Conclusions

Much work remains to be done in the field, as the potential bidirectional relationships within the DS-periodontitis-AD triad are currently under active investigation (Figure 1). However, the exact intersection among these three conditions currently lacks robust, longitudinal experimental and clinical validation, and no interventional clinical trials have yet demonstrated that periodontal therapy provides direct cognitive benefits in the DS population. Prospective studies tracking periodontal status and microbiological shifts alongside standardized cognitive monitoring are essential to clarify potential causal links. Given the prominent role of neuroinflammation in AD pathogenesis, secondary chronic conditions that promote the systemic over-secretion of pro-inflammatory mediators represent important modifiable risk factors to investigate. Approaching the DS-AD relationship beyond the traditional *APP* gene-dosage paradigm by integrating a neuroinflammatory standpoint —with particular emphasis on the *APO* ε4 isoform— offers novel insights into practical recommendations —such as early, lifelong screening and tailored prevention strategies— while simultaneously overcoming the profound socioeconomic, behavioral, and professional healthcare barriers inherent to this vulnerable population will be paramount to developing effective, translationally relevant therapeutic and preventive approaches.
